# Management of acute aortic dissection and thoracic aortic rupture

**DOI:** 10.1186/s40560-018-0287-7

**Published:** 2018-03-01

**Authors:** Toshihiro Fukui

**Affiliations:** Department of Cardiovascular Surgery, Kumamoto University Hospital, 1-1-1 Honjo, Chuo-ku, Kumamoto, 860-5663 Japan

**Keywords:** Acute aortic syndrome, Acute aortic dissection, Aortic aneurysm, Rupture, Stent graft

## Abstract

**Background:**

Both acute aortic dissection and ruptured aortic aneurysm are leading causes of death in cardiovascular disease. These life-threatening conditions have recently been categorized as acute aortic syndrome. This review describes the etiology, clinical presentation, and therapeutic options for acute aortic syndrome including acute aortic dissection and ruptured aortic aneurysm.

**Main body:**

Several diagnostic tools for detecting these critical conditions have been developed including computed tomography, ultrasonography, magnetic resonance imaging, and laboratory tests. Early and accurate diagnosis is most important to determine appropriate treatment. Initial treatment for these conditions should be aimed at controlling pain and the hemodynamic state with further treatment based on the imaging diagnosis and hematological assessment. Surgical outcomes after acute aortic syndrome are improving gradually; however, mortality remains high. Recently, thoracic endovascular aortic repair has become an alternative technique to treat complicated type B aortic dissection. Rapid treatment after early diagnosis is essential to save patients’ lives.

**Conclusions:**

Continuous advances in imaging and treatment technologies are improving short- and long-term outcomes in patients with acute aortic syndrome. Knowledge and interest in intensive care medicine in this area are contributing to improved outcomes, and further research into this life-threatening disease will lead to improvements in diagnosis and management.

## Background

Both acute aortic dissection and ruptured aortic aneurysm are leading causes of death in cardiovascular disease. These life-threatening conditions have recently been categorized as acute aortic syndrome. Acute aortic syndrome is defined as emergency conditions with similar clinical characteristics involving the aorta that include classical aortic dissection, intramural hematoma without intimal tear, penetrating atherosclerotic ulcer, and impending or ruptured aortic aneurysm [1]. Understanding the progression and extent of aortic disease is important because the treatment approach is highly dependent on the severity of aortic disease. Recently, several diagnostic tools have been developed to detect these critical conditions including computed tomography, ultrasonography, magnetic resonance imaging, and laboratory tests. Early and accurate diagnosis is essential to determine appropriate treatment. Initial treatment should be aimed at controlling pain and the hemodynamic state followed by considering the necessity and indications for surgical treatment by replacing the diseased aortic segment with an artificial graft. More recently, endovascular techniques and devices have been developed to treat aneurysms and dissections of the descending thoracic aorta even in acute settings.

In this article, we review the etiology, clinical presentation, and therapeutic options for acute aortic syndromes including acute aortic dissection and ruptured aortic aneurysm.

## Etiology, classification, and symptoms

### Aortic dissection

Acute aortic dissection is defined as dissection occurring within 2 weeks of onset of pain [2]. Subacute and chronic dissections occur between 2 and 6 weeks, and more than 6 weeks from the onset of pain, respectively [2].

**Fig. 1:**
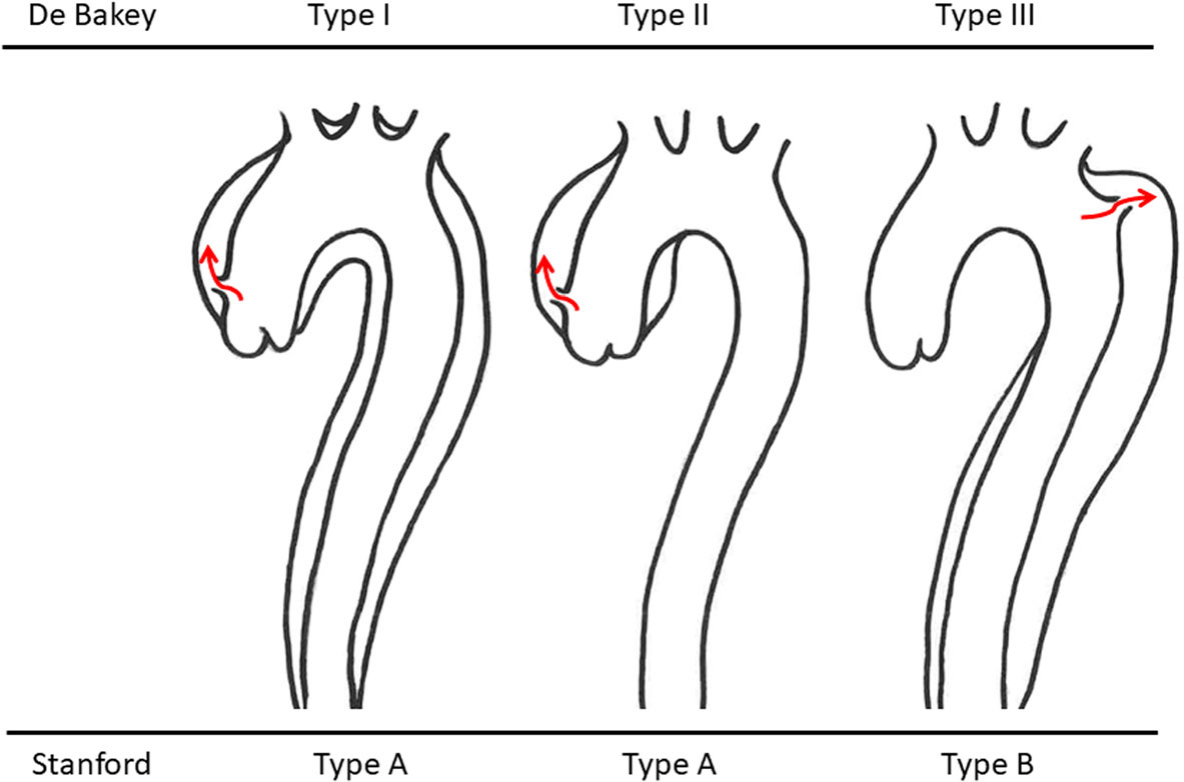
Classification of aortic dissection. De Bakey type and Stanford type are indicated

Two classifications are most commonly used for aortic dissection (Fig. 1). The DeBakey system is classified into three types (types I, II, and III) according to the site of the first entry of dissection [3]. Type I has the first entry in the ascending aorta and propagates distally to the descending aorta. Type II has the first entry in the ascending aorta and does not propagate to the aortic arch. Type III has the first entry in the descending aorta and propagates distally above (type IIIa) or below (type IIIb) the diaphragm. The Stanford system is classified into two types (types A and B) based on involvement of the ascending aorta [4]. Type A includes dissection in the ascending aorta regardless of the site of first entry. Type B does not include dissection in the ascending aorta.

The most common risk factor for aortic dissection is poorly controlled hypertension (65–75% risk with a history of hypertension [1, 5]). Other risk factors include age, male sex, smoking, pre-existing aortic diseases or aortic valve disease, family history of aortic diseases, history of cardiac surgery, direct blunt trauma, and the use of intravenous drugs (such as cocaine or amphetamines) [1, 2, 6].

Sudden onset of severe chest and/or back pain is the most typical symptom. The pain may be sharp, ripping, tearing, or knife-like and is typically different from other causes of chest pain; the abruptness of its onset is the most specific characteristic [1, 5]. Patients with type A aortic dissection commonly have anterior chest pain; those with type B more frequently have back pain [7]. Therefore, the initial symptoms most often reflect the location of the primary tear. The pain often migrates from the first point to other sites according to the extension of the aortic dissection [1]. In patients with complications in other organs caused by extension of the dissection, various symptoms may appear. Cardiac complications are most frequently observed in patients with type A dissection and can include aortic regurgitation, myocardial ischemia or infarction, and tamponade. Aortic regurgitation may accompany 40–75% of patients with type A aortic dissection [8–10]. Myocardial ischemia or infarction may be present in 10–15% of patients with type A aortic dissection secondary to compression or obliteration of the coronary arterial ostium [10]. Patients with cardiac complications most often present with heart failure and cardiogenic shock; neurological symptoms may occur with dissection of the carotid or vertebral arteries. The frequency of neurological symptoms in type A aortic dissection ranges from 10 to 40%, and in half of affected patients, symptoms are transient [1, 11]. Disturbance of consciousness is a possible neurological symptom in patients with type A aortic dissection, which can vary from somnolence to deep coma. Neurological status depends on the degree of reduced blood flow to the brain that results from cerebral malperfusion, hypotension, or distal thromboembolism. Mesenteric ischemia occurs in < 5% of patients with both type A and B aortic dissection [11]. Because abdominal pain is often nonspecific, the diagnosis of aortic dissection is difficult in these patients; however, the in-hospital mortality of patients with mesenteric malperfusion is reported to be almost three times as high as patients without mesenteric malperfusion (63 vs. 24%, respectively) [11].

### Ruptured aortic aneurysm

The histopathological feature of aortic aneurysm is degeneration of the medial layer of the aortic wall, which mainly comprises structural proteins such as collagen and elastin [12]. Subsequent dilatation occurs gradually from hemodynamic forces on the arterial wall as well as intrinsic changes in the composition of the arterial wall itself [12].

Risk factors for aortic aneurysm are nearly identical to those for atherosclerosis and include age, male sex, smoking, hypertension, obesity, dyslipidemia, chronic obstructive pulmonary disease, and family history.

The definition of aneurysm is a permanent, localized arterial dilation to more than 50% of the normal diameter. In general, the descending aorta grows faster (3 mm/year) than the ascending aorta (1 mm/year) [13].

**Fig. 2:**
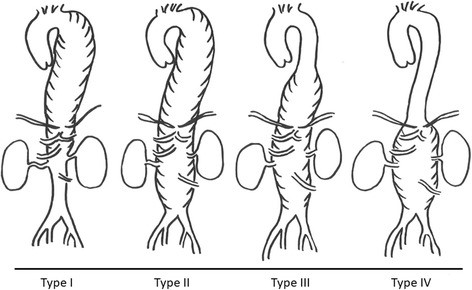
Classification of thoracoabdominal aortic aneurysm

The classification of aortic aneurysm is usually focused on the location of the aneurysm. The diaphragm separates an aortic aneurysm into a thoracic or abdominal aneurysm; however, a thoracoabdominal aortic aneurysm extends beyond the diaphragm. Thoracic aortic aneurysm is further categorized according to the location (ascending, arch, and descending) because the surgical method and approach for each location is completely different. Crawford and Coselli classified thoracoabdominal aortic aneurysms based on the extent of the aneurysm [14] (Fig. 2). Type I involves the descending thoracic aorta from the origin of the left subclavian to the suprarenal abdominal aorta; type II involves the descending thoracic aorta from the subclavian to the aortoiliac bifurcation; type III involves the distal thoracic aorta to the aortoiliac bifurcation; and type IV involves the abdominal aorta below the diaphragm. This classification system is convenient and is, therefore, frequently used. The surgical method and approach for each location differs; the outcomes also differ, and type II has the worst outcomes [12].

Sudden onset of severe chest and/or back pain is the most typical symptom associated with aortic dissection [1]. In patients with free-wall rupture, rapid hypotension may lead to deep coma or death; erosion or sealed rupture into the lung or esophagus can result in hemoptysis or hematemesis [15, 16]. Rarely, an ascending or arch aneurysm ruptures into the pericardial cavity and causes tamponade with cardiogenic shock [17]. Abdominal pain may be present in patients with a ruptured thoracoabdominal aneurysm; however, in patients with a contained or impending rupture, the hemodynamic state is stable because bleeding is stopped by the surrounding tissue or organs. Fewer than half of all patients with rupture arrive at the hospital alive; mortality may be as high as 54% at 6 h and 76% at 24 h after the initial event [18].

## Diagnosis

Initial diagnosis is extremely important in patients with acute aortic syndrome [2]. When a patient has sudden abrupt chest or back pain, imaging diagnosis should be the first concern, with simultaneous laboratory tests, including a biochemical study and complete blood count, and electrocardiogram. It is especially important to note the level of D-dimers in patients with acute aortic dissection [19–21]; a high level of D-dimers is immediately observable compared with other diseases [19]. The levels of other biomarkers (e.g., matrix metalloproinase-9 and transforming growth factor-beta) are increased in patients with acute aortic dissection. Increased concentrations of matrix metalloproinase-9 occur within 1 h of the onset of acute aortic dissection and remain elevated during 2 months’ follow-up [22]. Transforming growth factor-beta may be a surrogate biomarker to assess aortic expansion after dissection and could be used to predict the risk of rupture and the need for repair [22].

### Computed tomography

Computed tomography (CT) is the most reliable diagnostic tool in patients with acute aortic dissection or ruptured aortic aneurysm. Today, CT is available in most emergency rooms and can be performed rapidly [6, 22]. CT clearly demonstrates the site, location, size, and extent of the aortic aneurysm and clearly shows the relationship between the aneurysm and neighboring organs or vessel branches. Reconstructed 3-D imaging helps guide the surgical approach to the aneurysm.

**Fig. 3:**
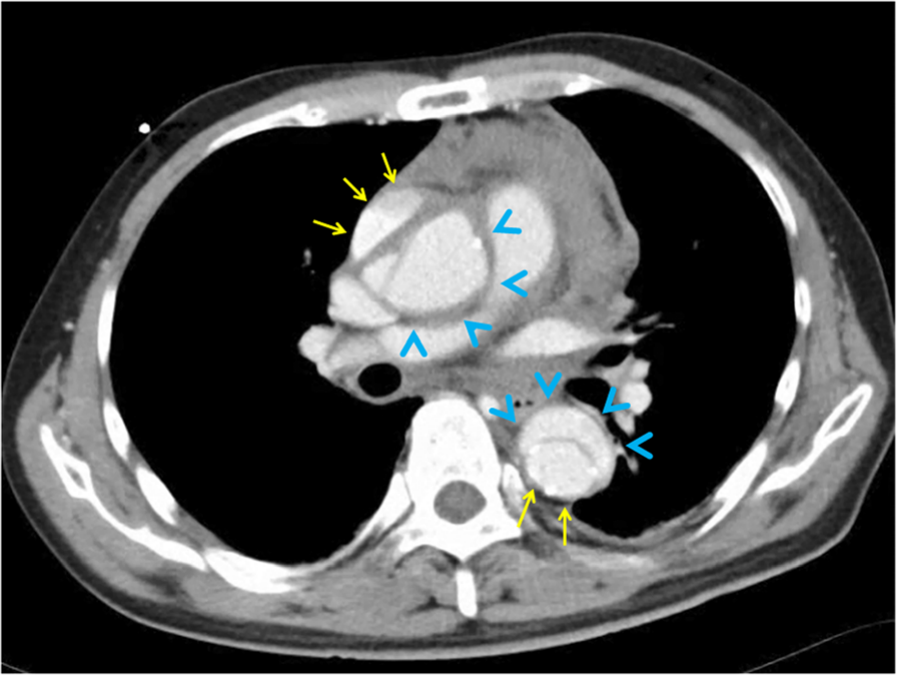
Computed tomography with contrast enhancement in a patient with acute aortic dissection. Arrows indicate the true lumen and arrowheads indicate the false lumen

In patients with acute aortic dissection, plain CT is useful for assessing inward displacement of intimal calcification [23]. Fluid effusion in the pleural cavity or pericardial space can also be identified using plain CT; however, contrast-enhanced CT is usually used to make a final decision for diagnosis and management in patients with acute aortic dissection [1, 24]. An intimal flap in the aorta divides the lumen in two (true and false lumens) (Fig. 3). A flap is present in the ascending aorta in patients with type A aortic dissection, and no flap is present in those with type B. The size of the false lumen is greater than that of the true lumen in most cases; however, in patients with intramural hematoma, aortic wall crescentic thickening can be seen extending distally and longitudinally [25]. In patients with aortic dissection, accurate discrimination between the true and false lumen is important to clarify which branches are perfused exclusively by the false lumen. It is also important to know whether the iliac and femoral arteries are dissected because the surgical approach, including endovascular treatment, is affected by dissection.

**Fig. 4:**
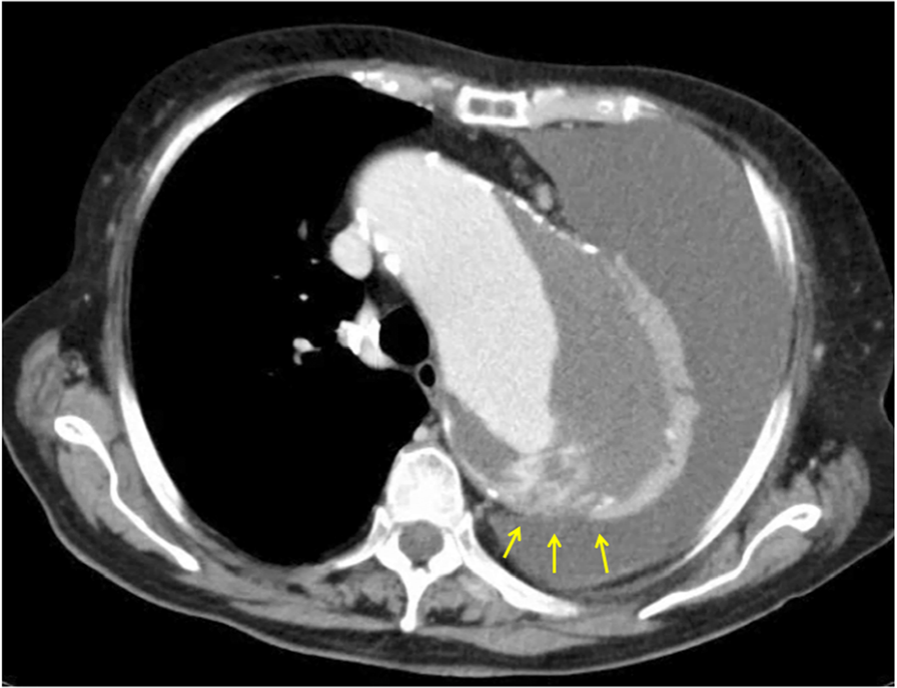
Computed tomography with contrast enhancement in a patient with aortic rupture. Arrows indicate the rupture site

In patients with suspicion of aortic rupture, plain CT is also useful to detect fluid effusion in the pleural cavity or pericardial space, and information regarding whether a hematoma exists around the aneurysm is useful in making the diagnosing aortic rupture. In such cases, CT with contrast media should be performed to detect the presence of contrast leaks (Fig. 4). Even with clear findings of hematoma around the aneurysm, contained or impending rupture of an aortic aneurysm is an indication for urgent treatment because of the risk of subsequent rupture [1]. Once acute aortic dissection is confirmed, it is also important to know the condition and quality of the iliac and femoral arteries because the surgical approach, including endovascular treatment, is affected. To perform endovascular treatment, anatomical factors, including the presence of adequate proximal and distal landing zones for the prosthesis, should be assessed simultaneously [26].

### Transesophageal echocardiography (TEE)

Transthoracic echocardiography (TTE) is less invasive compared with other imaging diagnostic modalities; it is portable and can be useful in an emergent situation. However, TTE has limitations in the narrow window obtained by the probe because of the bones and lungs, and it is operator-dependent [27]. TEE provides good image quality and has a wider window that includes the aortic arch and descending aorta, compared with TTE. However, TEE is invasive compared with TTE, and the patient requires some sedation. TEE also has a blind spot confined to the proximal arch because of bronchial air [28]. Both TTE and TEE provide information about the heart valves, ascending aorta, and aortic root, as well as cardiac function, and pleural and pericardial effusion can be checked quickly.

In most patients with acute type A aortic dissection, a flap and false lumen in the ascending aorta can be detected [29]; entry sites can also be detected by color Doppler imaging. Using TEE, entry sites in the arch or descending aorta are well visualized, and coronary obstruction resulting from the dissection can also be detected. In some patients, because acute aortic valve regurgitation may be associated with proximal aortic dissection, the function of the aortic valve should be checked; other valves are minimally affected by acute aortic dissection, directly. Intraoperatively, TEE is useful to assess the function of the heart and valves, as well as the condition of the false lumen, which can vary during the procedure.

In patients with ascending aortic rupture, TTE is useful to detect hematoma and effusion in the pericardial cavity. Also, the size of the sinus of Valsalva and ascending aorta can be measured using TTE, and the function of the aortic valve and other valves can be evaluated at the same time. TEE provides good information regarding the intra-aortic condition of the descending and thoracoabdominal aorta, which is useful when considering the surgical approach.

### Magnetic resonance imaging

Magnetic resonance imaging (MRI) is considered an accurate diagnostic tool for detecting acute aortic syndrome (sensitivity and specificity, 98%) [30]. MRI produces high-resolution aortic imaging with 3-D; however, it is rarely performed in emergency settings because of several limitations [6], including that it is not available in most emergency departments and it is inconvenient with a relatively long imaging acquisition time. MRI also cannot be performed in patients with claustrophobia, or in those with pacemakers, aneurysm clips, or other metal devices. Magnetic resonance angiography is used for patients who are allergic to the iodinated contrast agents used in CT or as a second diagnostic tool when CT is inadequate or the true diagnosis remains uncertain.

In patients with acute type A aortic dissection, identification of the intimal flap on MRI remains the key finding, usually seen first on spin-echo “black-blood” sequences [31]. The true lumen shows a signal void, whereas the false lumen shows higher signal intensity indicative of turbulent flow [32]. Flow in the false lumen and true lumen can be quantified using phase contrast cine-MRI or by tagging techniques. MRI is also useful to detect the presence of pericardial effusion, aortic regurgitation, or carotid artery dissection. MRI is seldom performed in patients with ascending aortic rupture because it is difficult to monitor unstable hemodynamic patients during imaging. However, intra-aortic conditions, including mural thrombi, are clearly visualized by MRI, and this information may help to determine the treatment strategy.

## Management and treatment

### Aortic dissection

Regardless of whether acute aortic dissection is type A or B, medical therapy to control pain and hypertension is essential in all patients. Beta blockers have the desired effect of reducing blood pressure and heart rate to the normal range [2]. These medications also protect the myocardium against ischemia. For most patients, systolic blood pressure should be controlled between 100 and 120 mmHg with a heart rate of approximately 60 bpm [2, 6]. Otherwise, vasodilators such as calcium channel blockers (nicardipine or diltiazem) or nitroglycerin are useful in reducing hypertension in an emergent situation.

In patients with type A aortic dissection, surgical treatment is the gold standard; mortality is 50% within the first 48 h if surgery is not performed [1]. However, early mortality after operation remains high at 9–25% [33–35]. Although surgical results are still unsatisfactory, long-term outcomes after operation are obviously better compared with medical therapy [36]. The aim of surgery in patients with type A aortic dissection is to prevent aortic rupture and pericardial tamponade and to relieve aortic regurgitation. Another goal is to improve blood flow in the branches disturbed by the false lumen. De Bakey et al. established basic surgical techniques including (1) excision of the intimal tear, (2) obliteration of entry into the false lumen, and (3) reconstitution of the aorta with interposition of a synthetic graft with or without reimplantation of the coronary arteries [37]. When the primary intimal tear is located in the ascending aorta, the ascending aorta is replaced. When the primary intimal tear is located in the aortic arch, both the ascending aorta and aortic arch are replaced. Aortic root replacement is performed when there is a tear in the sinus of Valsalva, and a valve-sparing operation may be an option if the aortic valve is normal or near normal [38]. However, valve-sparing procedures increase surgical time compared with composite graft replacement with an artificial valve. In some patients with myocardial ischemia or infarction because of coronary artery dissection or compression by a false lumen, coronary artery bypass grafting should be added [39]. Outcomes in patients with left coronary artery involvement are worse than in those with right coronary artery involvement.

There are several approaches to establishing cardiopulmonary bypass for surgery for type A aortic dissection [40]. In most emergent situations, the femoral artery and vein are used for cannulation. These vessels are convenient, and cannulation is simultaneously performed with opening of the chest. Otherwise, the subclavian or axillary artery, the apex of the heart, or the directly ascending aorta is chosen based on the surgeon’s preference. Adjunctive measures such as profound hypothermic circulatory arrest, retrograde perfusion, and selective perfusion of the head vessels are used for open distal anastomosis. The results of selective perfusion of the head vessels are better than for other methods regarding mortality and cerebral complications in patients undergoing aortic arch replacement [41].

Certain serious complications can occur after operation for type A aortic dissection. The incidence of stroke after operation for type A aortic dissection has been reported at 2–16% in recent studies [42]. Preoperative shock status is largely related to the incidence of postoperative stroke. Other major complications include renal failure, spinal cord injury, mediastinal bleeding, chylothorax, and mediastinitis. The incidence of each complication is institution- and surgeon-dependent and generally ranges from 3 to 10%, in our experience.

In most patients with type B aortic dissection, medical therapy including analgesia, antihypertensive drugs, and bed rest is performed. However, complicated type B aortic dissection, such as descending aortic rupture, uncontrolled pain, and malperfusion of the aortic branch or lower extremities, is an indication for urgent surgery [1]. The aim of surgical repair in patients with type B aortic dissection is to resect the primary entry tear and to replace the dissected descending aorta, which increases blood flow to the true lumen and improves organ ischemia. In this situation, the descending aorta is approached through a left posterolateral thoracotomy with cardiopulmonary bypass using the femoral artery and vein; deep hypothermic arrest is adopted for open proximal anastomosis. This operation in patients with type B aortic dissection has several potential complications including stroke, spinal cord ischemia, acute lung injury, and acute renal failure [43, 44]. More recently, thoracic endovascular aortic repair (TEVAR) has become an alternative technique to treat complicated type B aortic dissection [26]. The main goal of TEVAR is closure of the primary entry tear in the descending aorta. Blood flow is redirected into the true lumen, leading to improved distal perfusion by resolving malperfusion of visceral or limb arteries. Another aim of TEVAR is to stabilize the dissected aorta to prevent late complications by inducing aortic remodeling. Thrombosis of the false lumen may result in shrinkage and prevent aneurysmal degeneration; however, TEVAR remains limited in complicated cases because of a lack of evidence.

### Ruptured aortic aneurysm

Maintaining the hemodynamic condition of patients with free aortic rupture is difficult. The majority of patients with aortic rupture and continuous bleeding cannot survive even with massive transfusion and large doses of catecholamine. However, the hemodynamic status of patients with a contained rupture is relatively near stable, although blood transfusion should be started as soon as possible after the diagnosis is established. Traditionally, patients with this condition have been treated by open repair. Total arch replacement with median sternotomy is performed when an aneurysm is located in the arch or distal arch segment. If the descending aortic aneurysm is accompanied by an arch aneurysm, hybrid procedures (combined endovascular and vascular surgery procedures) are used [45, 46]. Hybrid procedures include a staged approach and a simultaneous approach. The staged approach involves total arch replacement with an “elephant trunk” at the first operation, and TEVAR is performed at some interval of days afterward. An elephant trunk is a hanging prosthetic graft in the descending aorta with only its proximal end anastomosed to the descending aorta and the four-branched arch graft. TEVAR is easily and safely performed with a good proximal landing zone at the elephant trunk. The simultaneous approach involves total arch replacement using an open stent graft, which is called the frozen elephant trunk procedure [47]. A prosthetic graft with a stent is manually inserted into the descending aorta during circulatory arrest. After insertion and deployment of the stent graft, the proximal side of this graft is anastomosed to the proximal end of the descending aorta and the four-branched arch graft. Spinal cord injury is a major complication following the frozen elephant trunk procedure [47] with a reported rate of up to 24% [47]. A distal landing zone lower than T7, prolonged lower body arrest time, and low arterial pressure have been suggested risk factors for spinal cord ischemia [48].

Rupture or contained rupture of a descending and thoracoabdominal aortic aneurysm is an indication for emergent or urgent operation. Surgery is traditionally performed through a left posterolateral thoracotomy with cardiopulmonary bypass. Deep hypothermic arrest is adopted for open proximal anastomosis if the appropriate proximal clamp site is unavailable. Usually, a straight prosthetic graft is used for descending aortic replacement; however, recently, endovascular repair has emerged as an alternative treatment option for suitable patients with ruptured descending aortic aneurysm [1]. In patients with a thoracoabdominal aortic aneurysm, a prosthetic graft with side branches for visceral arteries is used for repair. In some institutions, endovascular repair is used for a thoracoabdominal aortic aneurysm [49]; however, open surgical repair remains the standard treatment. Spinal cord injury is the most serious complication. Preoperative insertion of spinal drainage is not always used in emergent operations; however, it can be postoperatively inserted when the patient has spinal cord ischemia. Somatosensory-evoked or motor-evoked potentials for evaluating spinal cord ischemia, hypothermia, maintaining increased arterial blood pressure, and appropriate distal perfusion with cardiopulmonary bypass may protect against spinal cord injury [38].

## Conclusions

Acute aortic syndrome, including acute aortic dissection and ruptured aortic aneurysm, is the leading cause of death in cardiovascular disease. However, continuous advances in imaging and treatment technologies are improving short- and long-term outcomes. Also, knowledge and interest in intensive care medicine in this area are contributing to improved outcomes. Further research into this life-threatening disease will lead to improvements in diagnosis and management.

## Abbreviations

CT: Computed tomography
MRI: Magnetic resonance imaging
TEE: Transesophageal echocardiography
TEVAR: Thoracic endovascular aortic repair
TTE: Transthoracic echocardiography
